# Supplementary material for: What I don’t know can hurt you: Collateral combat damage seems more acceptable when bystander victims are unidentified
Source: PLoS One. 2024 Oct 23;19(10):e0298842. doi: 10.1371/journal.pone.0298842 (PMC11498727; doi:10.1371/journal.pone.0298842)
Supplement: S1 File — Supplemental materials including S1-S4 Tables full text from all conditions in all studies, additional measures: Studies 1a, 1b, 1c, 2, qualitative coding categories for bombing justifications Study 1b, replications and pilot studies, total war questionnaire items, additional study, Also, S1 Fig. Rating of firing acceptability in Study 1a. Ratings are split by condition and range from 1 (Not at all) to 7 (Very Much). Error bars represent a 95% confidence interval. Asterisks denote Dwass-Steel-Critchlow-Fligner pairwise comparison p-values compared with the unidentified bystander condition (*p < .05, **p < .01, ***p < .001). (PDF) [file pone.0298842.s001.pdf]

Table S1. Full text from all conditions in Studies 1a, 1b, 1c, Study 2 and Study 5. All conditions across studies 1a, 1b, 1c, Study 2, and Study 5 open with the same passage describing an ongoing war between ISIS and the United States followed by a story about a pilot tracking an ISIS operative who must choose whether to allow the operative to escape or fire a missile that would kill the operative but also kill a bystander. Conditions vary the subsequent description of the bystander.

Initial passage in all conditions:

The Middle East has seen a lot of armed conflict over the past few decades, especially in areas such as Iraq and Syria. The political vacuum created by these conflicts has allowed militant groups to gain power. ISIS is currently the most powerful militant Islamic terrorist organization in the area, and they are claiming territory and actively plotting attacks against America and other developed nations.

America and her allies have been conducting regular airstrikes over ISIS territory in an attempt to limit the expansion of ISIS and prevent it from mounting serious attacks. One day, a U.S. fighter jet is patrolling an area in Northern Iraq known to be controlled by ISIS. Intelligence has identified an ISIS operative in the area who is a prominent leader and military strategist for ISIS. The jet makes visual contact with this individual. He is wearing ISIS gear and carrying a rifle. Before the pilot can do anything, the ISIS operative notices the jet overhead and runs down a road into a nearby farmhouse.

Subsequent passage in each Study and Condition:

Study	Condition	Description of bystander
1a, 1b, 1c*	ISIS Soldier	Intelligence knows another ISIS soldier is living in the farmhouse the operative runs into. The soldier is staying in the house during operations in the area. He has supported ISIS in taking control of the region.
	Extremist Muslim	Intelligence knows a local civilian farmer is living in the farmhouse the operative runs into. The farmer has lived there his whole life raising goats for market. He is not a member of ISIS and has never provided them with support. However, he is a devout Muslim, and he appreciates the emphasis that ISIS places on fundamental Islamic beliefs and prays with them daily.
	War profiteer	Intelligence knows a local civilian farmer is living in the farmhouse the operative runs into. The farmer has lived there his whole life raising goats for market. He is not a member of ISIS and has never provided them with support. However, his small farm business has benefitted substantially from ISIS activity.
	Conflicted Muslim	Intelligence knows a local civilian farmer is living in the farmhouse the operative runs into. The farmer has lived there his whole life raising goats for market. He is not a member of ISIS and has never provided them with support. He feels deeply conflicted about ISIS. He is very upset about some things ISIS has done, but he also appreciates other things ISIS has done.

	Anti-ISIS Muslim	Intelligence knows a local civilian farmer is living in the farmhouse the operative runs into. The farmer has lived there his whole life raising goats for market. He is not a member of ISIS and has never provided them with support. He is a devout Muslim, but he is appalled by ISIS's violent interpretation of fundamentalist Islam.
	Innocent civilian	Intelligence knows a local civilian farmer is living in the farmhouse the operative runs into. The farmer has lived there his whole life raising goats for market. He does not support ISIS, but ISIS has taken control of the region.
	Unidentified	Intelligence knows a second individual is in the farmhouse but they don't know anything about that person.
Study 2	Percentages 0-100 in 10% increments summing to 100	Intelligence knows a second individual is in the farmhouse but they don't know anything about that person. Intelligence about ISIS in the region says that there is a [X] percent chance the second individual is also an ISIS operative and a [Y] percent chance they are an innocent civilian.

*Study 1c presented only the unidentified bystander condition

Table S2. Full text from all conditions in Study 3.
Study 3 manipulates details of the combatants in the war. The entirety of the vignette is included for each condition below.

Study	Condition	Full vignette
Study 3	Real-world conflict	The Middle East has seen a lot of armed conflict over the past few decades, especially in areas such as Iraq and Syria. The political vacuum created by these conflicts has allowed militant groups to gain power. ISIS is currently the most powerful militant Islamic terrorist organization in the area, and they are claiming territory and actively plotting attacks against America and other developed nations.<br><br>America and her allies have been conducting regular airstrikes over ISIS territory in an attempt to limit the expansion of ISIS and prevent it from mounting serious attacks. One day, a U.S. fighter jet is patrolling an area in Northern Iraq known to be controlled by ISIS. Intelligence has identified an ISIS operative in the area who is a prominent leader and military strategist for ISIS. The jet makes visual contact with this individual. He is wearing ISIS gear and carrying a rifle. Before the pilot can do anything, the ISIS operative notices the jet overhead and runs down a road into a nearby farmhouse.<br><br>Intelligence knows a second individual is in the farmhouse but they don't know anything about that person. The pilot can see both individuals in the house using infrared technology. The jet is low on fuel and must shortly return to base. The pilot must decide

		whether to attack the farmhouse with a missile, killing both people inside, or whether to exit the area, allowing both people to live.
	Fictional Conflict	The regions around the nation of Sorovia have seen a lot of armed conflict over the past few decades. The political vacuum created by these conflicts has allowed militant groups to gain power. The Sorovia Federation is currently the most powerful military organization in the area, and they are claiming territory and actively plotting attacks against Nibia and other developed nations.<br><br>Nibia and its allies have been conducting regular airstrikes over territory controlled by the Sorovia Federation in an attempt to limit the expansion of the Federation and prevent it from mounting serious attacks. One day a Nibian fighter jet is patrolling an area in Northern Sorovia known to be controlled by Federation forces. Intelligence has identified a Sorovia Federation operative in the area who is a prominent leader and military strategist for the Federation. The jet makes visual contact with the individual. He is wearing Federation gear and carrying a rifle. Before the pilot can do anything the Federation operative notices the jet overhead and runs down a road into a nearby farmhouse.<br><br>Intelligence knows of a second individual in the farmhouse but they don't know anything about that person. The pilot can see both individuals in the house using infrared technology. The jet is low on fuel and must shortly return to base. The pilot must decide whether to attack the farmhouse with a missile, killing both people inside, or whether to exit the area, allowing both people to live.

Table S3. Full text from all conditions in Study 4.
Study 4 manipulates the nationality (Iraqi, Swedish) and the guilt (innocent reporter, ISIS reporter) of the bystander. The beginning passage is identical to that of Table S1.

Study	Condition	Description of Bystander
Study 4	Local, Innocent	Intelligence knows a **local Iraqi reporter named Omar Abbas** is living in the farmhouse the operative runs into. This reporter writes articles informing the world about ISIS activity and has lived in Iraq his whole life. **He does not support ISIS and never has,** but ISIS has taken control of the region. Using the jet's infrared technology, the pilot can see both the reporter and operative in the house.
	Foreign, Innocent	Intelligence knows a **foreign reporter from Sweden named Sven Gustafsson** is staying in the farmhouse the operative runs into. This reporter travelled from Sweden to Iraq to write articles informing the world about ISIS activity and is staying in the house during operations in the area. **He does not support ISIS and never has**, but ISIS has taken control of the region. Using the jet's infrared technology, the pilot can see both the reporter and operative in the house.

Local, Guilty	Intelligence knows a **local ISIS reporter named Omar Abbas** is living in the farmhouse the operative runs into. This reporter writes articles in support of ISIS and their ideology and has lived in Iraq his whole life. **He has consistently supported ISIS in taking control of the region.** Using the jet's infrared technology, the pilot can see both the reporter and operative in the house.
Foreign, Guilty	Intelligence knows a **foreign ISIS reporter from Sweden named Sven Gustafsson** is staying in the farmhouse the operative runs into. This reporter traveled from Sweden to Iraq to write articles in support of ISIS and their ideology and is staying in the house during operations in the area. **He has consistently supported ISIS in taking control of the region.** Using the jet's infrared technology, the pilot can see both the reporter and operative in the house.

Above descriptions were all proceeded by:

The jet is low on fuel and must shortly return to base. The pilot must decide whether to attack the farmhouse with a missile, killing both people inside, or whether to exit the area, allowing both people to live.

Study 1a: Additional Measures

Acceptability of Firing. As in Study 1a participants were asked "How acceptable is it for the pilot to fire upon the building, killing both people inside?" and answered on a Likert scale from 1 (*Not at all*) to 7 (*Very much*). A Kruskal-Wallis ANOVA observed significant differences between the vignettes, $\chi^2(6,456)=84.98$, $p<.001$, $\varepsilon^2=0.18$ (Figure S1). Dwass-Steel-Critchlow-Fligner pairwise comparisons showed the unidentified bystander (M=4.03, SD=1.82) was rated significantly higher than the innocent civilian (M=2.64, SD=1.78, W=6.02, p<.001), the anti-ISIS Muslim (M=2.69, SD=1.52, W=-5.99, p<.001), and the conflicted Muslim (M=2.69, SD=1.90, W=-5.85, p<.001). Additionally, the unidentified bystander was rated significantly lower than the ISIS soldier (M=5.18, SD=1.80, W=5.15, p=.005).

Figure S1. Rating of firing acceptability from 1 (Not at all) to 7 (Very much) in Study 1a split by bystander condition. Error bars represent a 95% confidence interval. Asterisks denote Dwass-Steel-Critchlow-Fligner pairwise comparison p-values compared with the unidentified bystander condition (*p<.05, **p<.01, ***p<.001).

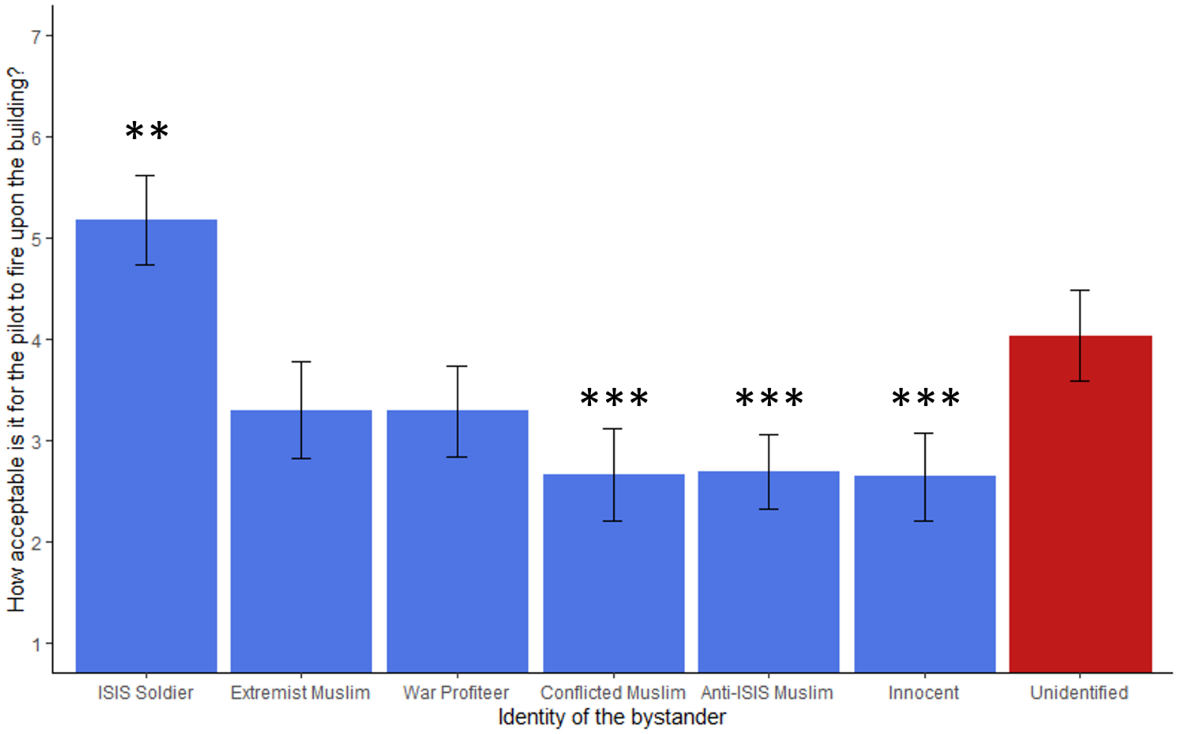

Comfort with Decision. Participants were asked "How comfortable are you with your decision?" and answered with a Likert scale between 1 (*Not very*) and 7 (*Very*). A one-way ANOVA showed no significant difference how comfortable people were with their answer across conditions, $F(6, 457)=1.42$, $p=.206$. However, an exploratory analysis found an effect of participant's decisions to fire: those who opted not to fire were significantly more comfortable with their decision overall ($M=5.38$, $SD=1.75$) than those who opted to fire ($M=4.63$, $SD=1.96$), $F(1,362)=17.4$, $p<.001$.

Deservingness to Die. As a manipulation check, participants were asked to rate the target (the ISIS operative) and the bystander on how much they deserved to die. These were answered on a scale from 1 (*Not at all*) to 7 (*Very much*). No matter which vignette the participant read, there was consensus that the ISIS operative was rated high in deservingness to die, $F(6,203)=0.80$, $p=.82$ ($M=5.08$, $SD=1.87$). Conversely, ratings of bystander deservingness to die varied significantly across condition, $F(6,199)=41.59$, $p<.001$, following the same pattern as firing acceptability and firing endorsement. Interestingly, just as the unidentified bystander saw a high endorsement of firing, participants also rated the unidentified bystander as relatively high on deservingness to die (Figure 2 in main text).

Military experience. Because of the relevance of formal military training to the dilemma in this study, we asked participants "Are you or is anyone close to you (i.e., close friends or family members) a member or Veteran of the U.S. Military?" Participants answered with one of seven choices including "I am currently in the US military," "I am a US military veteran," "I have never been in any military but I have close friends or family members who served in the Military (or are currently serving)," and "I have never been in any military and I do not have close friends or family members in any military either." Few participants in our sample had direct military experience. Only 22 out of 464 (4.7%) answered "I am currently in the US military," "I am a US Military veteran," or "I am currently in the military of a country that is not the US." To achieve comparable groups, we combined these items into a binary dependent measure. Participants who answered "I have never been in any military and I do not have close friends or family members in any military

either" (no military ties, *N*=154, 33.2%) were compared to those who gave any answer which indicated military ties (military ties, *N* = 310, 66.8%).

A generalized linear model using a log likelihood ratio test showed those with military ties were overall significantly more likely to endorse firing to kill both people (*M*=.432, *SD*=.496) compared to those with no military ties (*M*=.331, *SD*=.472), χ^2(1, 464)=4.30, *p*=.038. Additionally there was a significant interaction between military ties and the identity of the bystander, χ^2(6, 464)=16.25, *p*=.012, however, Bonferroni corrected *p* values for post hoc comparisons round to *p*=1.00 in a majority of pairwise comparisons due to the number of comparisons. Overall, we find a weak but significant effect across conditions whereby those with military ties tend to endorse firing the missile across conditions. However, because we could not obtain an adequate sample of participants with direct military experience, this data should be considered exploratory.

Religion. Participants reported their religion. The list of possible answers was Christianity (*N*=270, 58% of sample), Judaism (7, 2%), Hinduism (3, 1%), Islam (5, 1%), Buddhism (13, 3%), and Other (161, 35%). Five participants (1%) did not answer this question. Because there were so few religions besides Christianity represented, a large enough sample size could not be obtained for adequate comparisons. As a result, comparisons used the item of religiosity.

Religiosity. Participants rated their religiosity from 1 (*Not at all religious*) to 7 (*very religious*). Close to half our sample (42%) answered the lowest value for "not at all religious." To better fit our sample, we computed a binary variable for religiosity for participants that answered 1 ("Not at all religious") or any number higher than 1. A generalized linear model for logistic regressions showed that across conditions, those who reported no religiosity tended to endorse firing less (*M*= .327, *SD*=.470) than those who reported some amount of religiosity (*M*= .451, *SD*=.499), χ^2(1,464)=8.48, *p*=.004. The interaction between bystander identity and religiosity was nonsignificant, χ^2(1,464)=5.75, *p*=.452. Overall those who rated some amount of religiosity endorsed firing the missile at slightly higher rates across conditions compared to those who reported no religiosity. The pattern of judgment for bombing each bystander was the same for the religious and non-religious.

Other demographics**.** In Study 1, our sample consisted of 194 males, 269 females, and 1 other gender. A chi-squared test of independence showed no gender differences in rates of endorsing firing the missile, χ^2(2,464)=1.78, *p*=.411. The average age in our sample was 36.7 years (*SD*=12.0). A binary logistic regression showed age had no significant effect on endorsing firing the missile, *z*=1.15, *p*=.25.

Study 1b: Additional Measures

Acceptability of Firing. As in Study 1a participants were asked "How acceptable is it for the pilot to fire upon the building, killing both people inside?" and answered on a Likert scale from 1 (*Not at all*) to 7 (*Very much*). A Kruskal-Wallis ANOVA observed significant differences between the vignettes, χ^2(6)=54.91, *p*<.001, ε^2=.20. Pairwise comparisons showed ratings of acceptability of firing on the unidentified bystander were lower than those of the ISIS solider (*W*=4.38, *p*=.032), and higher than the anti-ISIS Muslim (*W*=-5.37, *p*=.003). All other comparisons with the unidentified bystander were non-significant (*p*<.05). The pattern of acceptability matched the pattern of endorsing firing in Study 1 (Figure 1 in main text) but many comparisons did not reach significance possibly due to the smaller sample size and lower power.

Comfort with Decision. Participants were asked "How comfortable are you with your decision?" and answered with a Likert scale between 1 (*Not very*) and 7 (*Very*). Contrary to study 1a, where was an overall effect of bystander identity on judgments of

comfort, $\chi^2(6)=16.62$, $p=.011$, $\varepsilon^2=.06$. Pairwise comparisons showed significant differences between conditions of the conflicted Muslim bystander (M=4.38, SD=1.69) and the innocent bystander (M=5.65, SD=1.58) ($W=4.78$, $p=.013$) and the conflicted Muslim (M=4.38, SD=1.69) and the anti-ISIS Muslim (M= 5.56, SD=1.55) ($W=4.46$, $p=.027$). No other pairwise comparisons were significant at the $p=.05$ level.

Deservingness to Die. Participants were asked to rate the target (the ISIS operative) and the bystander on how much they deserved to die. These were answered on a scale from 1 (*Not at all*) to 7 (*Very much*). As in Study 1a, there were no differences between vignette $\chi^2(6)=5.19$, $p=.52$, $\varepsilon^2=.02$, with high agreement that the ISIS operative deserved to die ($M=5.43$, $SD=1.48$). As in Study 1a, ratings of bystander deservingness to die varied significantly across condition, $\chi^2(6)=66.37$, $p=.001$, $\varepsilon^2=.25$. Pairwise comparisons showed that the unidentified bystander was rating significantly lower than the conflicted Muslim (W=-4.26, P=.041), the innocent civilian (W=-4.53, p=.023), the anti-ISIS Muslim (W=-5.04, .007), and marginally less than the extremist Muslim (W=-4.17, p=.050). Additionally, the unidentified bystander was rated lower in deservingness to die compared to the ISIS soldier bystander ($W=5.11$, $p=.006$). Overall, this reflects the same pattern of results seen in study 1a.

Ties to military. Military background was coded as in Study 1a. A generalized linear model using a log likelihood ratio test showed those with military ties ($N=156$) were overall not more likely to endorse firing to kill both people ($M=.333$, $SD=.473$) compared to those with no military ties ($N= 115$, $M=.365$, $SD=.484$), $\chi^2(1, 272)=0.184$, $p=.668$. Additionally there was no significant interaction between military ties and the identity of the bystander, $\chi^2(6, 272)=0.768$, $p=.993$. This fails to replicate the finding from Study 1a in which those with ties to the military were overall more likely to endorse firing across conditions.

Table S4. Qualitative coding categories for bombing justifications in Study 1b. Examples are quotes from participants in response to the prompt: "Briefly explain your decision about whether the pilot should fire on the building."

Category Label	Description	Example
**Greater Good**	*Appeal to saving more people at the expense of few; utilitarian calculus*	"Killing the ISIS member may save more lives in the long run. Unfortunately, the other person may have to die to save other lives. I'm thinking of the net effect"
**Collateral Damage**	*Collateral damage is justified in war*	"Unfortunately, war comes with collateral damage."
**Self defence**	*The pilot has a right to defend himself*	"If he doesn't [fire], ISIS might shoot him down"
**Duty**	*It is the pilot's job or responsibility to fire*	"It is wartime and hard decisions must be made. If it means saving one American from being destroyed then yes they should strike the house."
**Appeal to innocence**	*It is wrong to kill innocents*	"The pilot should never harm innocent people."
**Killing is wrong**	*It is wrong to kill*	"I don't believe violence and destruction is acceptable under any circumstances."
**The war is unjustified**	*This war is not justified*	"USA should not be policing foreign countries and certainly should NOT be engage in extra-judicial assassinations."
**Property damage**	*Concern over unjustly damaged property*	"It isn't clear who exactly the farmhouse belongs to - it could be some innocent civilian's property."
**Need permission**	*The pilot does not have the right to choose, or should seek permission or advice*	"I don't think it's the pilot's call to make a decision on whether to fire or not."
**Ignorance**	*The desire for more information or the belief that unseen variables could cause a bad outcome*	"Without enough intelligence on the situation, there's no way to qualify whether or not the strike would endanger or kill innocent noncombatants."
**Reputation concerns**	*Concern that killing civilians will harm the war effort by losing support of the locals or the international community*	"It's not worth blowing up the farmers house just to get one ISIS member. You'll destroy whatever goodwill you have with the locals in the region."
**Other**	*Any other justification*	"You can capture the individuals that are within the building and imprison them as that seems to be a much more painful punishment that immediate death."
**No justification**	*Not a justification, but still demonstrates they understood the story*	"This is an ethical dilemma, so there's no right or wrong answer."
**Affiliation with ISIS**	*Belief that the bystander is affiliated with ISIS when they are not*	"The person might be a terrorist as well so it would be a good counter measure to do so."

Study 1c: Additional Measures

Endorsing firing. A two-proportion test showed that participants were divided approximately 50/50 between firing and not firing: 44 (47%) endorsed firing and 49 (53%) did not endorse firing. Neither was significantly different from 50% (p=.679).

Acceptability of firing. Participants reported "How acceptable is it for the pilot to fire upon the building, killing both people inside?" and answered on a Likert scale from 1 (*Not at all*) to 7 (*Very much*). The average answer (M=3.84, SD=1.71) was not significantly different from the midpoint in a one-sample t test ($t(92)$=-0.91, p=.365, *Cohen's d* =-0.09).

Comfort with decision. Participants were comfortable with their decision, with an average comfort rating of 4.61 out of 7 (SD=1.87) that was significantly above the midpoint ($t(92)$=3.17, p=.002, *Cohen's d*=0.33). There was no difference in ratings of comfort between participants who endorsed firing and those who did not ($t(91)$=1.45, p=.150, *Cohen's d*=0.30).

Deservingness to die. As in studies 1a and 1b, participants rated the ISIS operative high on deservingness to die (M=5.61, SD=1.53). The unidentified bystander was rated lower (M=2.99, SD=1.49) and was rated significantly below the midpoint ($t(92)$=-6.53, p<.001, *Cohen's d*=-1.68). These ratings are similar to those found in Study 1a.

Military ties. Similar to study 1a, our participant pool had few members with military experience (N=8, 8%). As in study 1a, we divided participants into those with military ties (N=58, 62%) and those with no military ties (N=35, 38%). Those with military ties were marginally more likely to endorse firing (N_{Fire}=32, 55%) than those without military ties (N_{Fire}=12, 34%) ($\chi^2(1,93)$=3.82, p=.051, *odds ratio*=2.36).

Study 2: Additional Measures

Comfort with Decision. We computed a linear regression of comfort with decision depending on the percent chance the bystander is in ISIS. This analysis showed that as likelihood increased, participants rated their comfort lower, $F(1)$=5.19, p=0.02, R^2=0.017. However, consistent with Studies 1 and 2, people who accepted firing were less comfortable with their decision (M=4.47, SD=1.90) than people who did not (M=5.48, SD=1.69), F(1, 227)=21.9, p<.001. Previous studies showed no difference in comfort with decision across condition, but the difference here may reflect the finding that firing rates were strongly related to perceived likelihood that the target may be in ISIS. A mediation analysis showed that comfort with decision carried significant indirect variance from the percent chance that the bystander is ISIS on willingness to fire, *estimate* = 4.45e-4, *SE* = 2.20e-4, 95% CI [1.30e-5, 8.76e-4].

Military ties. Only 14 (5%) of our sample had military experience, so as in Study 1, we computed the military ties variable. In our sample 129 participants (43%) had ties to the military while 168 (57%) had no military ties. Those with military ties were no more likely to endorse the bombing than those without military ties ($\chi^2(1,297)$=0.04, p=.845).

Replications and Pilot Studies

Pilot Study: Enemy and innocent conditions

A pilot study (N=165) was run prior to Study 1 which contained only the two most extreme conditions from Study 1: vignettes where the bystander was 1) an ISIS soldier or 2) an innocent civilian. This study used the same dependent measures as Study 1 and found similar results to Study 2 for the included conditions. Similar to Study 1, manipulating the identity of the bystander impacted participants' judgments of whether the pilot should fire. When the bystander was described as being an ISIS soldier participants were significantly more likely to say the pilot should fire (M=.753, SD=.434) than when the bystander was described as an innocent civilian (M=.200, SD=.403) ($\chi^2(1,165)$=50.4, p<.001). Judgements

were similar when participants were asked to rate the acceptability of the pilot firing on a Likert scale from 1 (*Not at all*) to 7 (*Very much*)**.** When the bystander was a civilian, participants found it significantly less acceptable than when they were another ISIS operative, (M=2.51, SD=1.64 and M=4.9, SD=1.82 respectively) ($t(163)=8.9, p<.01$). Participants in both conditions were equally comfortable with their decision (M=5.1 SD= 1.65 for the ISIS operative vignette, M=5.17, SD=1.89 for the innocent civilian vignette, $t(163)=.29, p=.996$). Participants in both conditions were unsurprisingly equal in their ratings of the ISIS operative's deservingness to die (M=5.54, SD=1.76 for the ISIS agent vignette, M=5.53, SD= 1.79 for the innocent civilian vignette, $t(163)=.06, p=.95$). As in Study 1, participants differed in how they rated the bystander's deservingness to die, with the ISIS soldier's ratings being higher (M=4.4, SD=1.84) than the civilian's (M=1.1, SD=.545, $t(163)=15.3, p<.01$).

Ties to military. Similar to Study 1, participants were grouped by whether they 1) were currently or previously in the military or had friends or family in the military or 2) had no military experience nor friends or family who had military experience. A generalized linear model using a log likelihood ratio test showed those with military ties were overall not more likely to endorse firing to kill both people (M=.469, SD=.502) compared to those with no military ties (M=.507, SD=.504), $\chi^2(1,165)=0.196, p=.658$. Additionally there was no significant interaction between military ties and the identity of the bystander, $\chi^2(6,165)=0.625, p=.429$.This result seems at odds with results from Study 1, which found those with ties to the military were significantly more likely to fire overall. This difference could be due to the smaller sample size of this pilot study and fewer conditions from Study 1.

This pilot study demonstrates the core finding of Study 1 that when people are faced with a choice that would result in a death as a second person, it matters who the second person is. People distinguish between the second person being an enemy combatant and an innocent civilian, and are less likely to endorse killing the civilian than the enemy. Also they find such an action less acceptable in the civilian case compared to the enemy combatant case.

Replication: Extremist and anonymous conditions

A limited replication of Study 1 was done as part of a larger study not reported here (N=242). This study included only the conditions of the unidentified bystander, and the extremist Muslim bystander. Endorsement of firing was not different regardless of whether the bystander was a Muslim Extremist (44.2% endorsed firing) or an unidentified person (46.5% endorsed firing) ($\chi^2(1,242)=0.125, p=0.724$). Similarly participant's responses were not different in ratings of acceptability of bombing ($F(1,234)=2.77, p=.097$), comfort with their decision ($F(1,239)=2.36, p=.126$), or ratings of how much the ISIS operative deserves to die ($F(1,239)=2.59, p=.126$). However, the unidentified bystander was rated significantly higher in deservingness to die (M=2.68, SD=1.53) compared to the extremist Muslim bystander (M=2.13, SD=1.56) ($F(1,235)=7.60, p=.006$). Overall, these findings replicate those of Study 1 for the conditions in which the bystander is a Muslim extremist and an unidentified person.

Study 4: Total War questionnaire (1-7, '*strongly disagree – strongly agree*')

Please answer how much you agree or disagree with the following statements

1. When two countries are at war, cities, factories, and hospitals should be legitimate targets for military strikes if disrupting them would help the war effort.
2. In war, militaries should never target non-combatant personnel or buildings for military strikes even if it is strategically advantageous.

3. When in times of war, a nation has a responsibility to use any and all weapons at its disposal to protect its people, and has no responsibility for the safety of the people of the enemy nation.
4. In war, everything and anything is fair game.
5. In the US's fight against terrorism in the Middle East, it is acceptable to target civilian factories, farms and infrastructure with military strikes if disrupting them would protect US troops and help win the war.
6. During war it is acceptable to bomb cities and other population centers in an enemy nation even if it results in no direct strategic military advantage.
7. During war it is acceptable to bomb cities and other population centers in an enemy nation if it results in a crucial strategic advantage to help end the war.
8. During war it is acceptable to carry out a crucial military strike if some civilians would die as an unavoidable side effect.
9. During war it is acceptable to carry out a crucial military strike if some civilians would die as an unavoidable side effect as long a more lives would be saved in the long run.
10. In times of war if a civilian organization like a factory, farm, or hospital in an enemy nation is aiding or supporting an enemy military, it should be considered a legitimate target for a military strike.

Additional Study

Study S1: Manipulating bystander proximity to combatant

Study 1c demonstrated that many participants judge an unidentified bystander in a combat zone as more likely to be an enemy combatant than an innocent civilian. Gestalt psychology describes the ways in which people automatically organize neutral information into sensible groups (Wagemans et al., 2012).

One principle is the principle of proximity: things which are close together spatially tend to be perceived as sharing other characteristics. For instance, we may assume two individuals sitting near each other in a park are more likely to be friends than strangers, solely due to their proximity. It could be that proximity to combatants makes unidentified bystanders appear similar to those combatants, explaining the high rate of assumption that unidentified bystanders are actually combatants. Another principle of gestalt psychology is similarity: things that are similar in one dimension are often thought similar in other dimensions. It could be that presenting unidentified bystanders and enemy combatants as similar in a superficial way might sway judgments toward thinking they are similar in their allegiance.

To test this, we presented participants with a similar dilemma to those used in Study 1a and manipulated gestalt cues of proximity and similarity when describing the combatants and the unidentified bystanders. We manipulated whether the bystander is described as being in the same building as the target or an adjacent building, and whether the target and bystander are depicted visually as two dots of the same or different colors. If people are using gestalt cues to infer the unknown bystander is a combatant, we expect participants are more likely to endorse bombing when the bystander is depicted as being in the same building as the combatant and represented as the same colored dot. We predicted lower endorsement of bombing when the gestalt cues imply differences: when the bystander is in an adjacent building and is represented as a different colored dot from the combatant. We expect to see this effect when the bystander is unidentified, but not when the bystander is known to be an innocent civilian. This study was pre-registered at: https://aspredicted.org/WRS_PYO

Method. We recruited 400 American participants on Amazon Mechanical Turk. A total of 451 answered some part of our survey and 62 were excluding for failing to complete the survey or failing an attention check question, leaving a final sample of 389.

This study had 4 conditions in a 2x2 design. Across conditions, a vignette describes a pilot who must decide whether to fire a missile that would kill an enemy combatant and a bystander. The bystander is either described as 1) an innocent civilian or 2) an unknown person. Additionally, the bystander is alternatively descried as 1) being in the same house as the combatant and is shown visually as a dot of the same color as the combatant or 2) being in a different house from the combatant and is shown visually as a dot of a different color as the combatant. Images in conditions where the bystander and combatant are described as being different houses show an aerial view of a duplex with a wall separating two houses. In conditions where the two are described as being in the same house, the image was digitally altered to show a single house. Dependent measures were identical to those used in Study 1a.

If gestalt cues of similarity and proximity create a perception that the unidentified bystander is a combatant, we expect a pattern of results like those shown in figure S2. We predict gestalt cues to have no impact on the civilian bystander because their allegiance is stated outright, but to cause the unidentified bystander to be bombed more when gestalt cues are similar compared to when they are different.

Table S5. Full text of four conditions in Study S1. Condition labels are in the left column, relevant changes in text between conditions are highlighted in yellow.

Innocent bystander, gestalt differences	The Middle East has seen a lot of armed conflict over the past few decades, especially in areas such as Iraq and Syria. The political vacuum created by these conflicts has allowed terrorist organizations and militant groups to gain power. ISIS is currently the most powerful militant Islamic terrorist organization in the area, and they are claiming territory and actively plotting attacks against America and other developed nations. America and her allies have been conducting regular airstrikes over ISIS territory in an attempt to limit the expansion of ISIS and prevent it from mounting serious attacks.<br><br>One day, a **U.S. fighter jet** is patrolling an area in Northern Iraq known to be controlled by ISIS. Intelligence has identified an **ISIS operative** in the area who has been **making chemical weapons**. The jet makes visual contact with this individual. He is wearing ISIS gear and carrying a rifle. Before the pilot can do anything, the ISIS operative notices the jet overhead and **runs down a road into a nearby farmhouse**. Intelligence knows a **local civilian farmer** is living in **the house next to the farmhouse** the operative runs into. Despite being in a different building, the farmer is close enough that **they would be also be hit by any attack** the pilot could make on the ISIS operative in the farmhouse. The farmer has lived there his whole life raising goats for market. He does not support ISIS, but ISIS has taken control of the region. The pilot can see both the farmer and operative in the houses using the jet's infrared technology. The jet is low on fuel and must shortly return to base. The pilot must decide whether to attack the farmhouse with a missile, killing both people, or whether to exit the area, allowing both people to live.<br><br>The figure below represents the farmhouse and other structures in the area. The **red dot** indicates the ISIS operative, the **blue dot** indicates the innocent civilian. The blast radius for the missile is indicated by the yellow ring. The pilot confirms that only those two people would be harmed by launching the missile.

	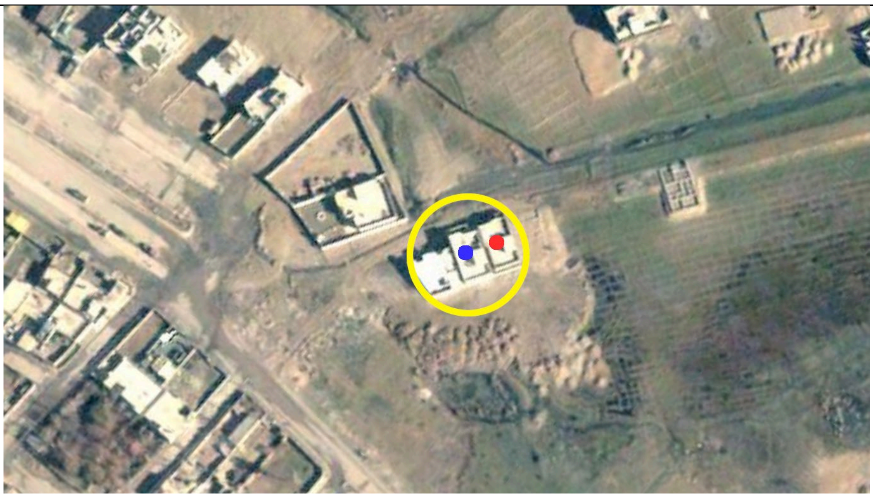
Innocent bystander, gestalt similarity	The Middle East has seen a lot of armed conflict over the past few decades, especially in areas such as Iraq and Syria. The political vacuum created by these conflicts has allowed terrorist organizations and militant groups to gain power. ISIS is currently the most powerful militant Islamic terrorist organization in the area, and they are claiming territory and actively plotting attacks against America and other developed nations.<br><br>America and her allies have been conducting regular airstrikes over ISIS territory in an attempt to limit the expansion of ISIS and prevent it from mounting serious attacks. One day, a **U.S. fighter jet** is patrolling an area in Northern Iraq known to be controlled by ISIS. Intelligence has identified an **ISIS operative** in the area who has been **making chemical weapons**. The jet makes visual contact with this individual. He is wearing ISIS gear and carrying a rifle. Before the pilot can do anything, the ISIS operative notices the jet overhead and **runs down a road into a nearby farmhouse**.<br><br>Intelligence knows a **local civilian farmer** is living in the **farmhouse** the operative runs into. Because he is in the same building, the farmer is close enough that **he would be also be hit by any attack** the pilot could make on the ISIS operative in the farmhouse. The farmer has lived there his whole life raising goats for market. He does not support ISIS, but ISIS has taken control of the region. The pilot can see both the farmer and operative in the house using the jet's infrared technology. The jet is low on fuel and must shortly return to base. The pilot must decide whether to attack the farmhouse with a missile, killing both people inside, or whether to exit the area, allowing both people to live.<br><br>The figure below represents the farmhouse and other structures in the area. The **right dot** indicates the ISIS operative, the **left dot** indicates the innocent civilian. The blast radius for the missile is indicated by the yellow ring. The pilot confirms that only those two people would be harmed by launching the missile.

<table>
<tr><td></td><td>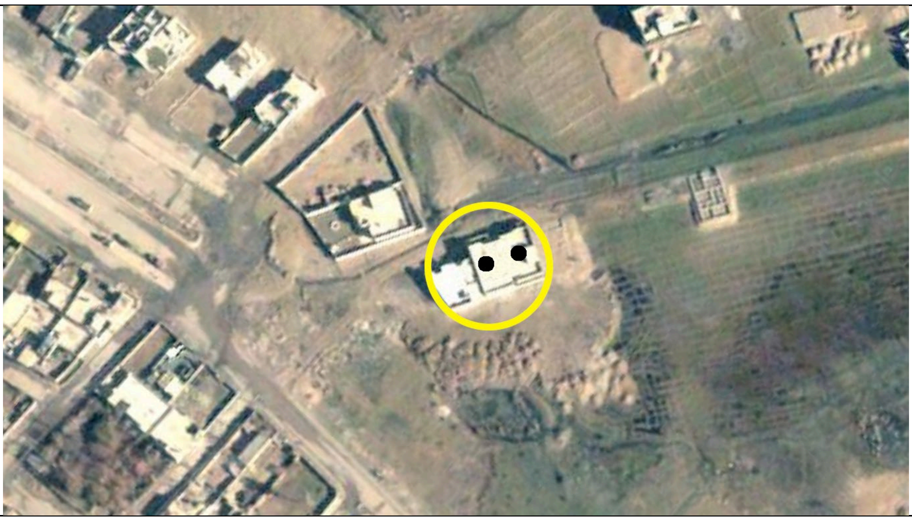</td></tr>
<tr><td>Unidentified bystander, gestalt difference</td><td>The Middle East has seen a lot of armed conflict over the past few decades, especially in areas such as Iraq and Syria. The political vacuum created by these conflicts has allowed terrorist organizations and militant groups to gain power. ISIS is currently the most powerful militant Islamic terrorist organization in the area, and they are claiming territory and actively plotting attacks against America and other developed nations.

America and her allies have been conducting regular airstrikes over ISIS territory in an attempt to limit the expansion of ISIS and prevent it from mounting serious attacks. One day, a **U.S. fighter jet** is patrolling an area in Northern Iraq known to be controlled by ISIS. Intelligence has identified an **ISIS operative** in the area who has been **making chemical weapons**. The jet makes visual contact with this individual. He is wearing ISIS gear and carrying a rifle. Before the pilot can do anything, the ISIS operative notices the jet overhead and **runs down a road into a nearby farmhouse**.

Intelligence knows a **second individual** is in **the house next to the farmhouse** but they don't know anything about that person. Despite being in a different building, the second individual is close enough that **they would be also be hit by any attack** the pilot could make on the ISIS operative in the farmhouse. The pilot can see both individuals in the houses using the jet's infrared technology. The jet is low on fuel and must shortly return to base. The pilot must decide whether to attack the farmhouse with a missile, killing both people, or whether to exit the area, allowing both people to live.

The figure below represents the farmhouse and other structures in the area. The **red dot** indicates the ISIS operative, the **blue dot** indicates the second individual. The blast radius for the missile is indicated by the yellow ring. The pilot confirms that only those two people would be harmed by launching the missile.
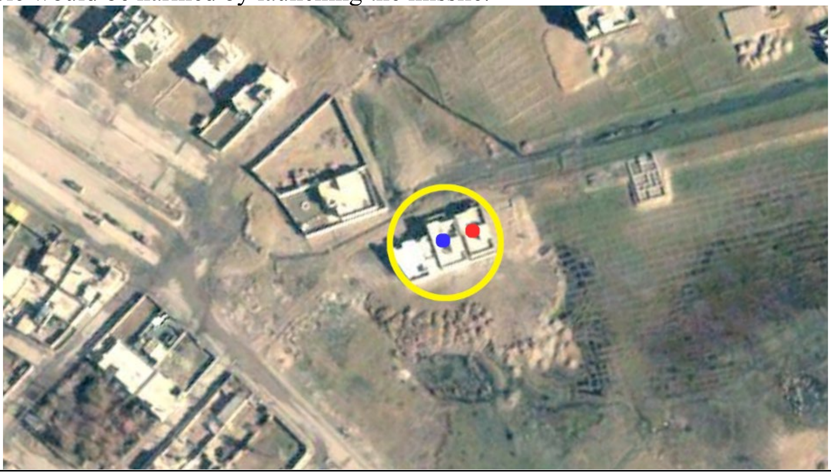</td></tr>
</table>

Unidentified bystander, gestalt similarity	The Middle East has seen a lot of armed conflict over the past few decades, especially in areas such as Iraq and Syria. The political vacuum created by these conflicts has allowed terrorist organizations and militant groups to gain power. ISIS is currently the most powerful militant Islamic terrorist organization in the area, and they are claiming territory and actively plotting attacks against America and other developed nations.<br><br>America and her allies have been conducting regular airstrikes over ISIS territory in an attempt to limit the expansion of ISIS and prevent it from mounting serious attacks. One day, a **U.S. fighter jet** is patrolling an area in Northern Iraq known to be controlled by ISIS. Intelligence has identified an **ISIS operative** in the area who has been **making chemical weapons**. The jet makes visual contact with this individual. He is wearing ISIS gear and carrying a rifle. Before the pilot can do anything, the ISIS operative notices the jet overhead and **runs down a road into a nearby farmhouse**.<br><br>Intelligence knows a **second individual** is in the **farmhouse** but they don't know anything about that person. Because they is in the same building, the second individual is close enough that **they would be also be hit by any attack** the pilot could make on the ISIS operative in the farmhouse. The pilot can see both individuals in the house using the jet's infrared technology. The jet is low on fuel and must shortly return to base. The pilot must decide whether to attack the farmhouse with a missile, killing both people inside, or whether to exit the area, allowing both people to live.<br><br>The figure below represents the farmhouse and other structures in the area. The **right dot** indicates the ISIS operative, the **left dot** indicates the second individual. The blast radius for the missile is indicated by the yellow ring. The pilot confirms that only those two people would be harmed by launching the missile.<br>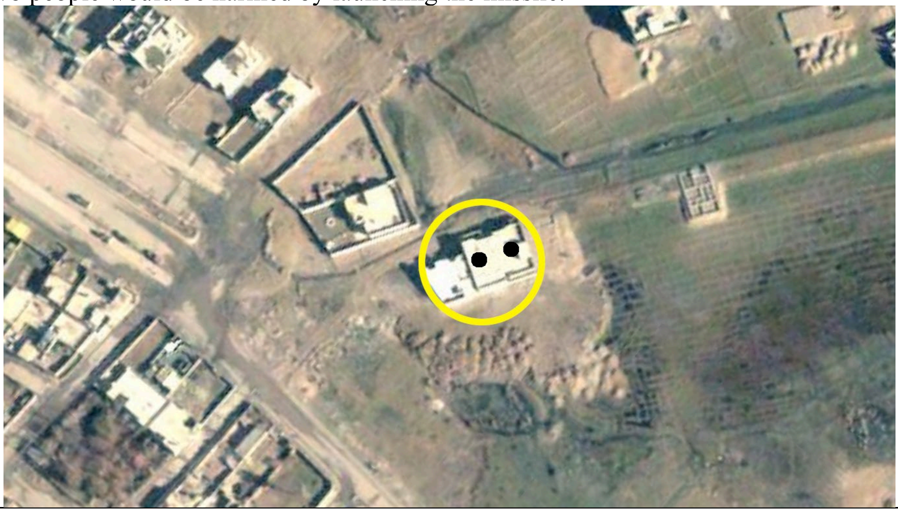

Figure S2. Predicted results supporting the hypothesis of Study S1.

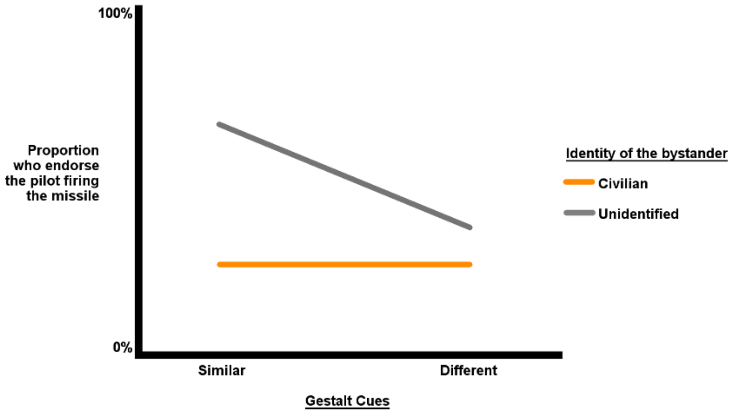

Results. We predicted an interaction between our variables of gestalt cues (similar vs different) and the identity of the bystander (innocent vs unidentified). Specifically, we predicted the unidentified bystander would be bombed at a higher rate when gestalt cues presented him as similar to the combatant compared to when cues presented him as different. We also predicted this effect would present in the civilian condition, hence the interaction. Replicating prior studies, there was a significant main effect of bystander, such that there was more endorsement of bombing the unidentified compared to the innocent bystander ($\chi^2(1, 389)=31.16$, $p<.001$, $OR=0.29$, 95%CI[0.19,0.45]). Against our hypothesis, when comparing bombing endorsement to gestalt cues and bystander identity, there was no significant interaction ($\chi^2(1, 389)=2.95$, $p=.086$, $OR=2.17$, 95% CI[0.90,5.31])), nor a significant main effect for similar vs different gestalt cues (($\chi^2(1, 389)=0.16$, $p=0.692$, $OR=0.91$, 95% CI[0.59,1.43]). However, the data do seem to trend in the predicted direction (Figure S3). Similarly, when measuring ratings of bombing acceptability, there was no significant main effect of gestalt cues ($F(1,385)=0.00$, $p=.978$, $\eta^2=0.00$), nor a significant interaction ($F(1,385)=1.33$, $p=.249$, $\eta^2=0.00$). Similar to previous studies, there was a significant main effect of bystander, such that there higher ratings of acceptability for bombing the unidentified compared to the innocent bystander ($F(1,385)=35.03$, $p<.001$, $\eta^2=0.08$).

Figure S3. Line graph for Study S1 comparing endorsing the bombing to gestalt cues (similar vs different) and bystander identity (innocent vs unidentified). Contrary to our hypothesis, there is no significant interaction, nor a significant main effect of gestalt cues. Error bars represented 95% CI.

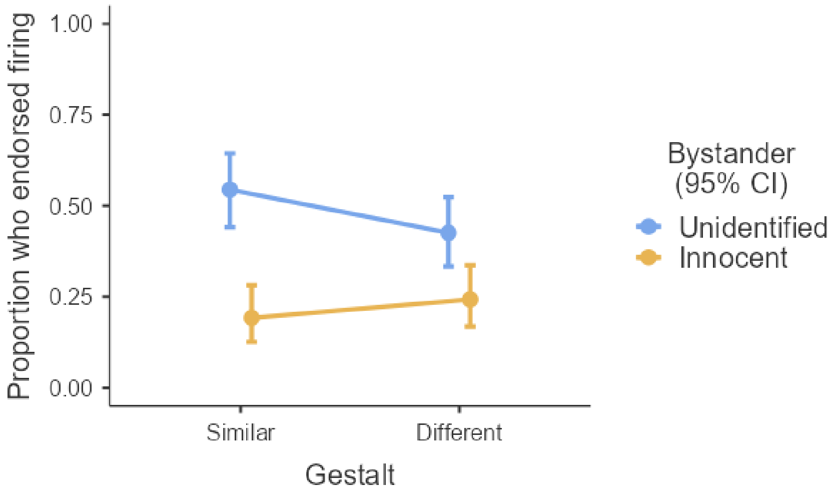

Discussion. Prior studies showed that people are likely to judge a bystander in a warzone to be a combatant rather than a civilian. One possible explanation for this is the gestalt cues of proximity (being near a combatant) and similarity (being of the same region, nation, culture, ect.). To explore this we presented two vignettes from Study 1a, which featured a bombing dilemma with either an innocent or an unidentified bystander caught in the crossfire. Here, we also manipulated gestalt cues to present the bystanders as more or less near the combatant (the gestalt cue of proximity) or more or less similar (the gestalt cue of similarity). Contrary to our hypothesis, our manipulation of gestalt cues produced no significant effects on either bombing endorsement nor ratings of bombing acceptability. This implies that cues like proximity or similarity of the unidentified bystander compared to the combatant cannot explain the high rates of bombing endorsement for unidentified bystanders.
