# Supplementary material for: What I don’t know can hurt you: Collateral combat damage seems more acceptable when bystander victims are unidentified
Source: PLoS One. 2024 Oct 23;19(10):e0298842. doi: 10.1371/journal.pone.0298842 (PMC11498727; doi:10.1371/journal.pone.0298842)
Supplement: Attachment — Submitted filename: Response to Reviewers.docx [file pone.0298842.s002.docx]

To the editors at PLOS ONE,

We thank you and the reviewers for their time and attention in reviewing this manuscript. Below addresses the changes made to the attached manuscript.

Response to Reviewer #1

To address the suggestion outlining the gap the research fills we added 5 lines to the second to last paragraph of the introduction explicitly addressing the research gap (lines 82-87). We hope this clarifies the contribution of this paper.

Along these same lines we also added a few points to the conclusion section of the general discussion to elaborate more on the practical implications of the research (lines 857-858, 862-863). This should make more obvious the meaning impact this research could have. We appreciate these suggestions and hope it will make the significance of this paper clear to the reader.

Response to Reviewer #2

As for the sources for lines 60-65 in the section about what Prospect Theory might predict about wartime judgments, we added two new citations (26 & 27) and two sentences of elaboration to further clarify (Lines 59-66).

For the source of lines 70-73, we have added a few lines of clarification that we are speculating about how belief in total war might predict wartime decision making. A thorough search of the empirical literature using multiple digital platforms found no empirical papers that assessed belief in total war as we describe it here. Thus, as to our knowledge, this is the first empirical paper to define and measure total war beliefs and how they apply to wartime decision-making. We make it clear that this is something that is unknown to set up how the findings of this paper fill this research gap (lines 74-77).

The reviewer also asks about inclusion or exclusion criteria about the participants’ ideas/prejudices regarding Islam and/or terrorism. We included everyone who took the survey so long as they gave complete data and passed competence check items. We did not measure or exclude participants based on prejudices of Islam or any other ideological or political beliefs. Though this is an interesting idea, and something we are considering in future studies, for an initial paper demonstrating our observed effects we wanted to cast as wide a net as possible. This was the case for all studies in the paper and we have added a note (lines 118-119) making that clear. This was also previously noted at the end of the discussion (lines 853-855).

We also proofread for copy editing and made a few minor changes to grammar. These can also be found in tracked changes in the attached document. Thank you all for the feedback and we appreciate the suggestions at improving this manuscript. We hope these edits are to your satisfaction and we welcome any further feedback.
